# Improving single pixel imaging performance in high noise condition by under-sampling

**DOI:** 10.1038/s41598-020-76487-3

**Published:** 2020-11-10

**Authors:** Fangyuan Sha, Sujit Kumar Sahoo, Huy Quoc Lam, Beng Koon Ng, Cuong Dang

**Affiliations:** 1 Centre for Optoelectronics and Biophotonics (COEB), School of Electrical and Electronic Engineering, The Photonics Institute (TPI), Nanyang Technological University Singapore, 50 Nanyang Avenue, Singapore, 639798 Singapore; 2 School of Electrical Science, Indian Institute of Technology Goa, At Goa College Engineering Campus, Farmagudi, Ponda, Goa 403401 India; 3 Temasek Laboratories @ Nanyang Technological University Singapore, 50 Nanyang Avenue, Singapore, 639798 Singapore

**Keywords:** Electrical and electronic engineering, Imaging techniques

## Abstract

Single-pixel imaging could be a superior solution for imaging applications where the detector array is very expensive or not even available. Sampling order, sampling ratio, noise and type of transforms affect the quality of the reconstructed image. Here, we compare the performance of single pixel imaging (SPI) with Hadamard transform (HT) and discrete cosine transform (DCT) in the presence of noise. The trade-off between adding image information and adding noise in each coefficient measurement results in an optimum number of measurements for reconstruction image quality. In addition, DCT shows higher image quality with fewer measurements than HT does. We then demonstrate our SPI with optimum sampling strategy for a large set of images and lab experiments and finally put forward a quality control technique, which is corroborated by the practical experiments. Our results suggest a practical approach for SPI to improve the speed and achieve the highest possible image quality.

## Introduction

Digital imaging has been based on a 2D detector array (or a 2D pixel array) that samples the intensity of the field at a regular interval in space^1,2^. Recently, there has been a rapid development of single-pixel imaging (SPI) technology^1,3–24^ which uses only one detector to sample the intensity of the field sequentially^17^. The sampling mechanism can be at the illumination side, while the detection measures the response of the scene for each illumination pattern, which is often referred to as computational ghost imaging. Another approach is to sample the image at the imaging plane using programable masks. The two schemes are mathematically the same and have been developed parallelly^25^. The cost reduction for detectors becomes obvious, especially in the case of imaging in ultraviolet or infrared spectrum where a detector array is very costly or even not available. Because a single bucket detector only measures the sum of light intensity, the signal can be very strong and the measurement can tolerate even highly scattering media between the patterned illuminated field and the detector^18^.

Because of the sequential sampling mechanism, we need a good solution to reduce time for measurement and calculation, which are all scaled by the resolution requirement (total pixel number) of the final image^12,26^. A straightforward way is to increase the sampling speed, which was demonstrated successfully with structured illumination using a high-speed LED array^27^. Different approaches have been also proposed to reduce the number of measurements. One can simply do under-sampling; and others do compressive sensing or even adaptive sensing. Compressive sensing uses random-based patterns to illuminate the object while the other approaches utilize orthogonal-based patterns. Significant achievements have been reported on reduction of measurements, shorter computational time, and improved imaging quality^5,8,12,14,22,28–32^. However, compressive sensing or adaptive sensing requires complicated algorithms which could cost even longer computational time than actual measurement time, and hence apparently is unacceptable in live high-speed imaging^8,12,14,22,32^. In addition, these algorithms are more sensitive to measurement noise. On the other hand, the calculation time for the under-sampling method is negligible. We can get comparable performance to compressive or adaptive sensing, and even better performance when we consider them in the same imaging time (measurement time + computational time). More importantly, by controlling the sampling ratio in the under-sampling approach, the quality control strategy for the SPI technique can be made a priory^3,18,19,33,34^.

Currently, orthonormal transforms used for under-sampling are usually Fourier transform (FT) and HT. Zhang and colleagues have given a comparison between HT and FT^19^. FT single-pixel imaging is more efficient for the under-sampling approach than HT single-pixel imaging (HT-SPI) because more signal energy concentrated at low frequency patterns for most images in real life. FTs generate complex coefficients, which leads to doubling the number of measurements. The image quality of the FT comes at the cost of significantly long measurement time and ruins the advantages of under-sampling. In contrast, DCT as an alternative to FT, is also introduced in SPI^23,35–43^. Similar to HT, DCT generate real coefficients, reducing the number of measurements significantly. A detailed comparison between HT and DCT for SPI in terms of image quality, noise robustness and the efficiency of measurement becomes important for real applications.

In this paper, we discuss the SPI technique in practical applications where the effects of noise and the illumination pattern’s bit-depth are present. When the signal to noise ratio (SNR) of each measurement is low, some of the measurements will add more noise than information for reconstruction. In this situation, it is unwise to improve the quality of a reconstructed image simply by increasing the amount of measurement until full sampling^13,17^. More specifically, we present a comparison between HT-SPI and DCT-SPI in terms of reconstruction image quality, noise robustness by both simulation analysis and lab demonstration. We also put forward a quality control method to increase efficiency of measurements, thus, reducing the measurement time without compromising the computational time.

## Single-pixel imaging with HT and DCT

Here we analysed SPI with HT and DCT by consideration of practical applications where illumination patterns, their bit-depth and measurement noise play important roles. For realistic pattern illumination (non-negative intensity), we add 1 in the transform basis. The inverse transform patterns can be generated by the following equation:

1 $$ P_{D} \left( {x,y} \right) = \frac{1}{2}\left[ {1 + D^{ - 1} \left\{ {\delta_{D} \left( {u_{0} ,v_{0} } \right)} \right\}} \right] $$

${D}^{-1}$ is the inverse transform and ${\delta }_{D}$ suits Eq. (6):

2 $$ \delta_{D} = \left\{ {\begin{array}{*{20}c} {1,u = u_{0} ,v = v_{0} } \\ {0,{\kern 1pt} \quad {\kern 1pt} {\kern 1pt} {\kern 1pt} {\kern 1pt} otherwise} \\ \end{array} } \right. $$

Sources of noise could be from environment, detectors and illumination sources affecting the signal quality directly. The bit-depth of illumination patterns indicates how precise the illumination source is in displaying the grey-scale patterns. Obviously, low bit-depth of illumination patterns would reduce the imaging quality of the DCT-SPI, while the HT-SPI based on binary patterns is immune to this effect. The bit-depth of analog to digital conversion (ADC) in the detector would play an important role in SPI as well; however, current technology can easily access very good detectors (more than 10-bit ADC). Here, we do not discuss the quantization of the detector and 12-bit and 10-bit ADCs are used in our simulation and lab demonstration, respectively.

### Quantization of illumination patterns

**Figure 1:**
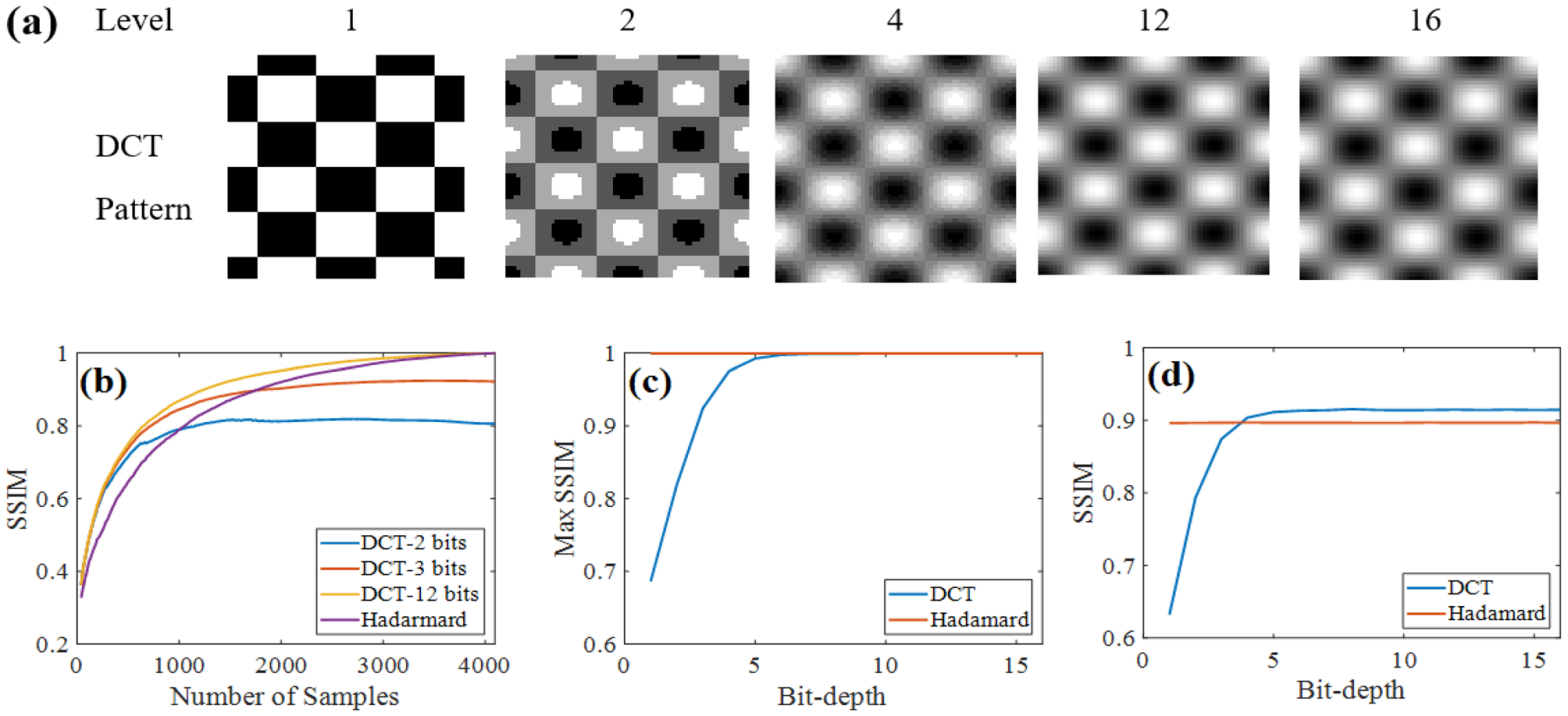
(**a**) Quantized patterns of DCT-SPI at different bit-depth level of 1, 2, 4, 12, 16 bits (**b**) SSIM as a function of sampling ratio at different quantization levels with measurement SNR of 40 dB. (**c**) SSIM at 100% number of measurements as a function of illumination pattern’s bit-depth (**d**) SSIM at 50% number of measurements as a function of illumination pattern’s bit-depth.

While HT only needs 1-bit for illumination patterns, DCT requires more bits because poor quantization will affect the orthogonality of the DCT patterns, leading to low reconstruction quality. Figure 1a shows the patterns of a typical DCT displayed by a projector at different quantization levels (from 1 to 16 bits). The higher the bit-depth is, the closer the pattern is to the expected DCT. The patterns for HT are always binary images, which are displayed similarly to the 1-bit pattern in Fig. 1a. We use the SSIM (structural similarity index) to describe the quality of the reconstructed image with respect to the original one (the ground truth)^44^. The value of 1 implies complete similarity or best reconstruction image. To make sure of the practicability of simulation, we add low noise with an SNR of 40 dB into the measurement signal. In addition, to having an accurate quality assessment of the reconstruction images, we select 12 images randomly from the USC-SIPI image database, convert to 64 × 64 pixel resolution as ground truth images for the experiment; then the SSIM scores of all reconstructed images are averaged.

Figure 1b shows the performance of HT-SPI and DCT-SPI at different quantization levels. For $64\times 64$ resolution, the full sampling number is 4096. The sampling process acquires the transform coefficients from low to high frequency patterns in sequence as more energy of natural images is concentrated in the low-frequency bands. When the bit-depth is too low (≤ 2 bits), the pattern digitalization noise dominates the information contribution of high frequency patterns, creating low quality images. With higher bit-depth (≥ 3 bits), the SSIM increases monotonically with a greater number of samples. Figure 1c presents the maximum SSIM of DCT-SPI as a function of the illumination pattern’s bit-depth in comparison to HT-SPI. With patterns of less than 3 bits, the maximum SSIM of DCT-SPI is lower than 0.8. Current display technology can easily achieve 8-bit grey scale display, for which the maximum SSIM for DCT-SPI could reach unity with full sampling number, similar to HT-SPI. However, as presented in Fig. 1b, the SSIM of DCT-SPI increases faster than that of HT-SPI at low frequency, i.e. the DCT-SPI achieves higher quality images than HT-SPI does at a low number of samples (sub-sampling). This implies that the DCT concentrates more information of an image than the HT does in low frequency patterns; while more information can be extracted with the HT for high frequency patterns. Figure 1d presents SSIM with only 50% sampling as a function of illumination pattern’s bit-depth. DCT-SPI has apparently higher SSIM as long as the pattern bit-depth is at least 4.

### Effect of noise and under-sampling solution for SPI

**Figure 2:**
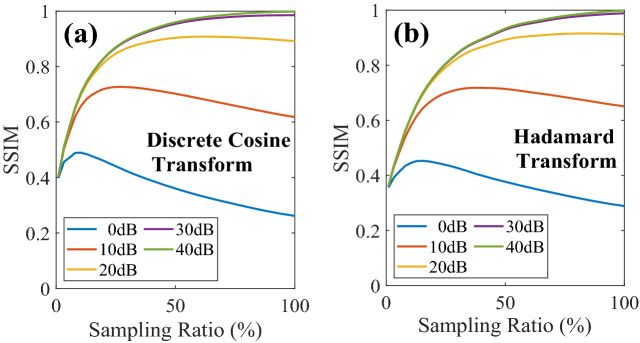
The quality of reconstructed images by DCT-SPI (**a**) and HT-SPI (**b**) as a function of the sampling ratio at different SNR.

Current 8-bit display technology can solve the digitalization noise of illumination patterns and allows DCT-SPI to achieve image quality equivalent to HT-SPI, which is immune from this noise. It is worth investigating the performance of SPI with other types of noise, which affect both HTs and DCTs. Figure 2 presents the image SSIM for both DCT-SPI and HT-SPI as a function of sampling ratio. Here, we use 8 bits for DCT illumination patterns. For high SNR (30–40 dB), the image information carrying in each illumination pattern will enhance image quality and the SSIM reaches maximum at full sampling for both DCT-SPI and HT-SPI. However, larger system noise (low SNR, typically less than 30 dB) has a more serious effect on the high frequency patterns where the noise can dominate the image information carried in each pattern.

Initially, measurements for low frequency patterns capture more information about image information; therefore, the SSIM sharply increases at first. Then it reaches a plateau, or even decrease for lower SNR. This implies that the measurements with high frequency patterns cannot capture more image information than noise. This observation is consistent in both DCT-SPI and HT-SPI. Therefore, we should take a lower number of measurements (under-sampling) for optimal image quality, which is an important contribution for practical applications.

**Figure 3:**
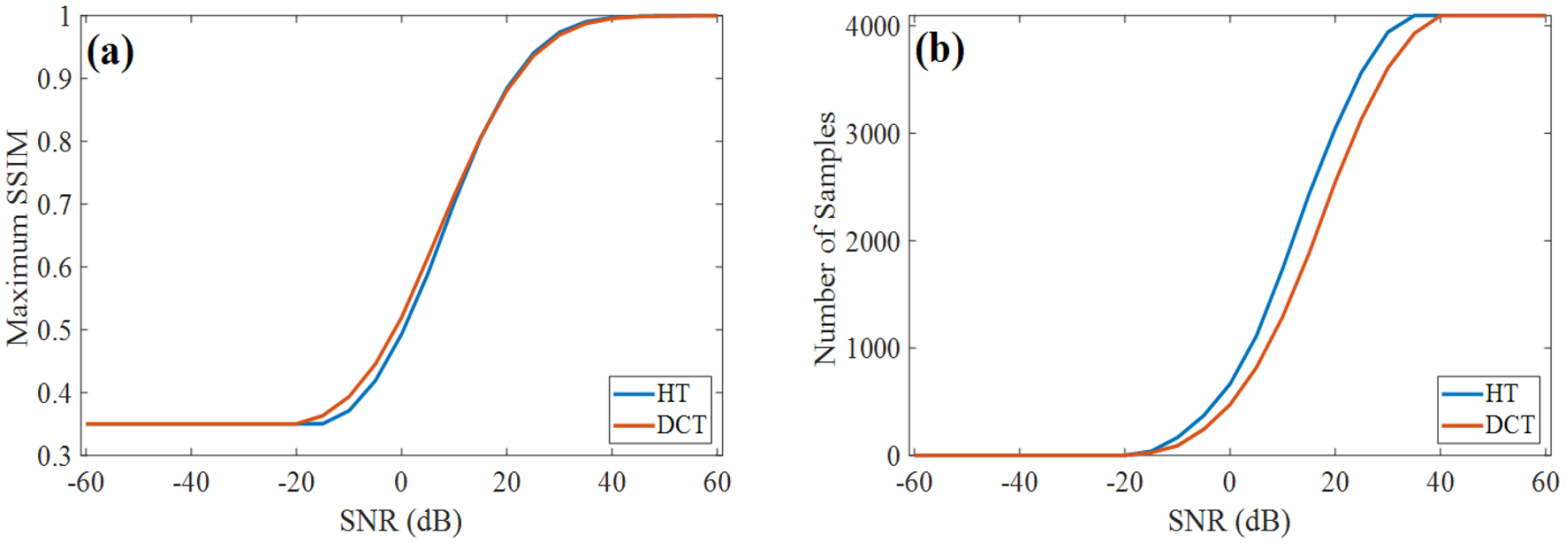
Performance of SPI with DCT and HT at different noise levels. (**a**) The dependence of maximum reconstruction SSIM on system SNR. (**b**) Sampling ratio to get the best reconstruction SSIM as a function of SNR.

However, because the DCT concentrates more image information in low frequency patterns, as discussed in Fig. 1b, we expect superior performance in low SNR compared to HT. Figure 3a shows the relationship between the system SNR and maximum SSIM, which is the highest SSIM of corresponding curve in Fig. 2. It is obvious for both DCT and HT that increasing SNR of measurement, the reconstruction quality will be better, resulting in higher achievable SSIM. When the noise is super-high (SNR < − 40 dB), both the DCT and HT do not really reconstruct the image, i.e. SSIM ~ 0. At the other extreme (noise level is very low, i.e. SNR > 10 dB), we do not see a noticeable difference of maximum SSIM between DCT-SPI and HT-SPI. In the low SNR region (from – 40 to 10 dB), the best achievable SSIM for DCT-SPI is slightly higher than that for HT-SPI. However, the most significant advantage of DCT-SPI is the sampling ratio that obtains the maximum reconstruction SSIM.

Figure 3b shows the sampling number to get the best reconstruction SSIM as a function of the system SNR. It is remarkable that the DCT requires less samples to get the best reconstruction performance compared to the HT for SPI for SNR ranging from – 20 to 40 dB. In other words, the DCT is more efficient in sample utilization than the HT. An important conclusion here is that DCT-SPI can reconstruct higher quality images with a fewer number of measurements in comparison with HT-SPI, especially for noisy conditions. This is a crucial point for SPI in gaining both image quality and frame rate.

### Validation of the under-sampling solution

**Figure 4:**
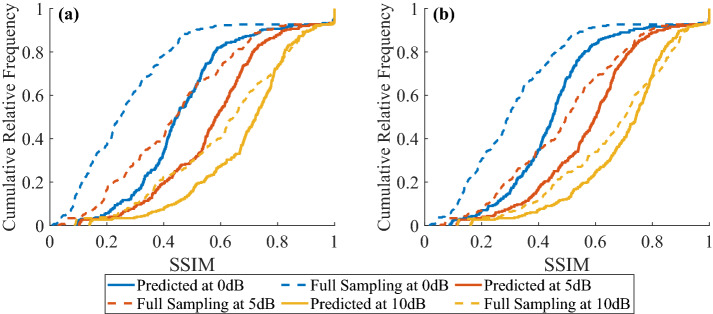
Cumulative relative frequency of SSIM by (**a**) DCT-SPI and (**b**) HT-SPI for different SNR.

Here, we would like to demonstrate our under-sampling strategy with SPI for a larger set of new images. We randomly selected 300 images from DIP4E Global Student Support Package^45^ for the test. Each image is sampled by the DCT and the HT at full sampling and optimum sampling with three different levels of SNR (0 dB, 5 dB and 10 dB). The optimum sampling number (474, 824, 1284 samples for DCT; 666, 1115, 1742 samples for HT) for each corresponding SNR (0 dB, 5 dB and 10 dB) is obtained from Fig. 3b. We show the cumulative relative frequency of SSIM by HT-SPI and DCT-SPI in Fig. 4. Apparently, SPI with optimum sampling numbers provides more images at higher SSIM than SPI with full sampling; and DCT-SPI at optimum sampling provides more images at higher SSIM than HT-SPI at optimum sampling. Beside the quality improvement, the advantage of frame rate is also obvious with optimum sampling compared to full sampling.

## Lab demonstration

**Figure 5:**
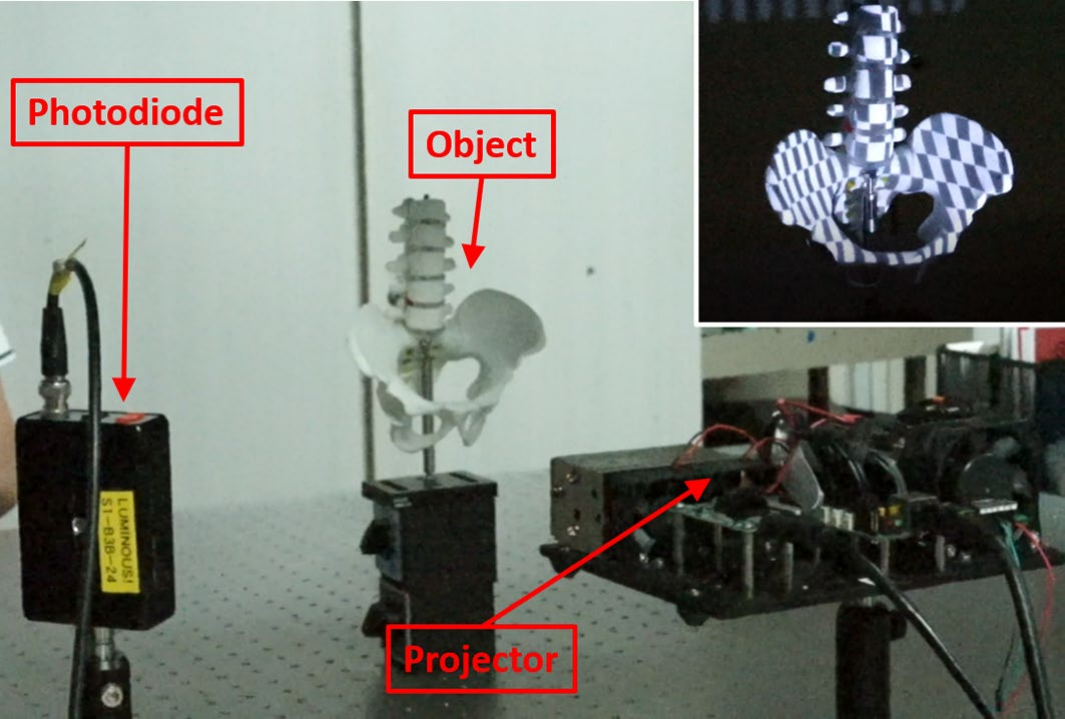
Experimental setup for SPI in the lab for real object imaging. Inset: The object is illuminated by a Hadamard pattern.

Here, we demonstrate the SPI with a projector and a photodetector in the lab. The signal from the photodetector is read by the data acquisition card (DAQ) and synced with the displayed images (Fig. 5). It is worth noting here that the commercial projector (EPSON LCD 430) is set with specific gamma curves from manufacturer to enhance displayed colours and contrast for human vision. Our displayed patterns are recalibrated to correct this gamma curve and achieve linearity for our SPI. After correction, our projector can display images at about 7-bit grey scale, which is sufficient for DCT-SPI to achieve image quality as good as HT-SPI.

After displaying each illumination patterns, we measure 3200 data points of scattered light intensity. Averaging all 3200 data points, we have the best measurement (i.e. the highest SNR: SNR_0_) for each illumination pattern. With full sampling (4096 measurements) and the highest SNR, we achieve the best reconstruction image which is considered as the ground truth for subsequence SSIM calculation. The image quality for DCT and HT is identical in this high SNR scenario; therefore, the calculated SSIM results do not depend on whether DCT or HT reconstruction result is chosen as the ground truth. We now randomly select N data points from 3200 original ones (N < 3200) for averaging then do image reconstruction. Because the SNR is proportional to N^0.5^, we can report image quality (SSIM) at different SNR relative to the SNR_0_ by choosing different values of N.

Table 1 shows the comparison results for DCT-SPI and HT—SPI with the real objects. The SSIM results are presented as the averaging SSIM for 10 different experimental objects, while the images are shown for a hipbone model. We can see that only 100 samples (2.5% full sampling number) can give the basic structure of an object for DCT-SPI, while HT-SPI needs at least 400 samples to visualize the object structure. At the same under-sampling conditions, the image reconstructed by DCT is better with higher SSIM than that by HT. DCT-SPI generates smoother images, while HT-SPI generates more-pixelated images as the results of binary illumination patterns. For high SNR such as SNR_0_ and SNR_0–9_, the images become better with increasing sampling number and both SPI methods reconstruct equivalently high image quality at full sampling of 4096 samples. However, in the low SNR scenario, the image quality does not always increase with more (or full) samplings. For example, at SNR_0–12_, the SSIM reaches the limit at around 1280 samples for DCT-SPI, the balance between gaining information and adding noise makes the image SSIM stable at approximately 0.61 for any greater numbers of sampling. While SSIM for HT-SPI monotonically increases with the sampling number. At SNR_0–15_, the SSIM is maximum at about 1130 samples for DCT-SPI and at about 2670 samples for HT-SPI, then drops at higher sample numbers because adding noise is more than gaining information for higher frequency patterns. The experimental results are consistent with our simulation results in Fig. 3. In such low SNR scenario, SPI could get the best results and highest frame rate by utilizing DCT with only 1000–1200 measurements.

**Table 1:**
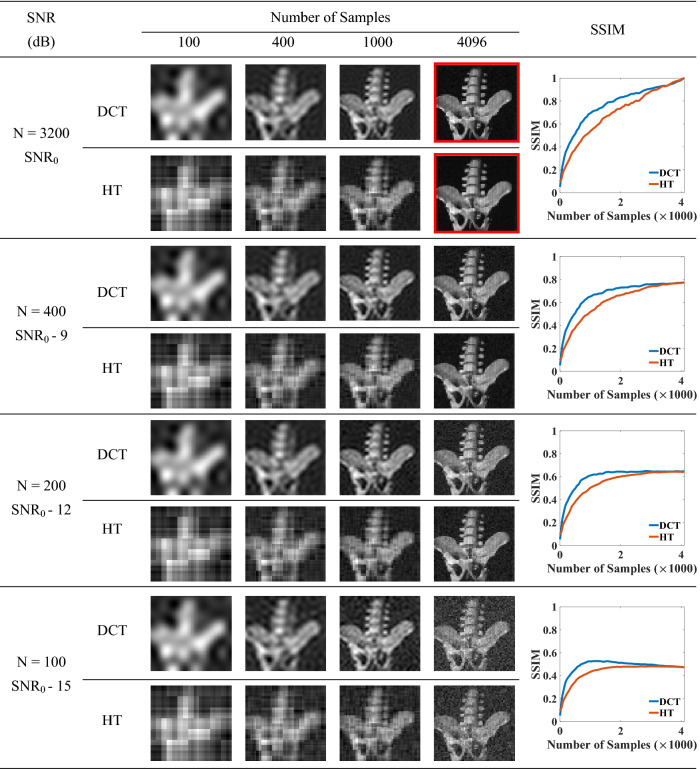
Comparison results for real objects.

The highest-quality images in the red boxes are identical and considered as the ground truth for subsequence SSIM calculation.

### Conclusion

We present a detailed comparison between the DCT and HT for SPI technique in both simulation and experiments. Quantization bit-depth is unique to DCT illumination patterns, which would cause some adverse effects if the bit-depth is less than 6 bits. However, current display technology with at least 8-bit grey scale does not affect the performance of DCT-SPI. The advantages of DCT-SPI are more obvious in the case of low SNR where the competition between imaging information and noise power in each illumination pattern plays the key role in image quality. The key message of this work is that the highest image quality does not necessarily need full sampling. The DCT is more efficient than the HT in carrying low frequency image patterns. DCT-SPI can achieve higher image quality with a smaller number of samples than HT-SPI, which is especially important for SPI performance for a low SNR system. More interestingly, our results suggest an optimum sampling number achieves the best image quality for a specific system SNR. This can save data acquisition (therefore increasing the imaging frame rate) and simultaneously produces the best SSIM.
